# Lymphocyte Subsets and Inflammatory Cytokines of Monoclonal Gammopathy of Undetermined Significance and Multiple Myeloma

**DOI:** 10.3390/ijms20112822

**Published:** 2019-06-10

**Authors:** Alessandro Allegra, Vanessa Innao, Andrea Gaetano Allegra, Marta Pugliese, Eleonora Di Salvo, Elvira Ventura-Spagnolo, Caterina Musolino, Sebastiano Gangemi

**Affiliations:** 1 Division of Hematology, Department of Human Pathology in Adulthood and Childhood “Gaetano Barresi”, University of Messina, 98125 Messina, Italy; aallegra@unime.it (A.A.); vinnao@unime.it (V.I.); andrea.allegra@hotmail.it (A.G.A.); martapugliese@libero.it (M.P.); cmusolino@unime.it (C.M.); 2 National Research Council of Italy (CNR)-Institute of Applied Science and Intelligent System (ISASI), 98164 Messina, Italy; 3 Legal Medicine Section, Department for Health Promotion and Mother-Child Care, University of Palermo, 90127 Palermo, Italy; elvira.ventura@unipa.it; 4 School and Division of Allergy and Clinical Immunology, Department of Clinical and Experimental Medicine, University Hospital “G. Martino”, Via Consolare Valeria SNC, 98125 Messina, Italy; gangemis@unime.it

**Keywords:** monoclonal gammopathy of undetermined significance, multiple myeloma, T lymphocytes, cytokine, alarmin, inflammation, immunosurveillance

## Abstract

Almost all multiple myeloma (MM) cases have been demonstrated to be linked to earlier monoclonal gammopathy of undetermined significance (MGUS). Nevertheless, there are no identified characteristics in the diagnosis of MGUS that have been helpful in differentiating subjects whose cancer may progress to a malignant situation. Regarding malignancy, the role of lymphocyte subsets and cytokines at the beginning of neoplastic diseases is now incontestable. In this review, we have concentrated our attention on the equilibrium between the diverse lymphocyte subsets and the cytokine system and summarized the current state of knowledge, providing an overview of the condition of the entire system in MGUS and MM. In an age where the therapy of neoplastic monoclonal gammopathies largely relies on drugs capable of acting on the immune system (immunomodulants, immunological checkpoint inhibitors, CAR-T), detailed knowledge of the the differences existing in benign and neoplastic forms of gammopathy is the main foundation for the adequate and optimal use of new drugs.

## 1. Introduction

### 1.1. General Considerations for MGUS

The term monoclonal gammopathy of undetermined significance (MGUS) designates the existence of a serum monoclonal protein in subjects with no sign of multiple myeloma (MM) or other related disorders, and represents the most frequent monoclonal gammopathy [1].

In MGUS, the amount of monoclonal Ig in the blood is <30 g/L, and monoclonal Ig accounts for 20%–70% of all Ig; thus, in MGUS, the formation of polyclonal Ig is conserved, though habitually decreased compared to healthy subjects. A relevant distinction between MGUS and MM is the finding that circulating monoclonal plasma cells are augmented in active MM. In fact, all subjects with MGUS had <3 × 10^6^ plasma cells per liter [2].

MGUS has been proposed to arise as an effect of the prolonged stimulation of B cells, possibly by microbial and self-antigens [3,4,5,6,7]. While this is generally believed to be benign, a fraction of MGUS subjects are connected with a significantly augmented risk for transformation into MM [8]. Although most MGUS never progresses towards smoldering myeloma (SMM) and MM, the possibility of progression of MGUS into SMM and MM, which are malignant diseases, is estimated at 1% per year per patient. Currently, there are no criteria in the diagnosis of MGUS that are helpful for differentiating between subjects whose disease will not evolve from those whose disease will progress to a malignant state [1].

### 1.2. MGUS Progression: Genetic and Microenvironment Factors

Numerous theories have been proposed to explicate MGUS progression; among these, one includes the achievement of genetic modifications, although no exclusive genetic modifications have yet been recognized to discriminate subjects with MGUS from those with MM. Karyotypic analysis in subjects with MGUS has been hindered by a low fraction of bone marrow plasma cells that are generally nonproliferating. Nevertheless, in a study that combined fluorescence in situ hybridization (FISH) with cytomorphology, chromosomes 3, 7, 11, and 18 were studied [9]. These chromosomes have been discovered to be aneuploid by FISH in MM. Three hybridization signs for one of these chromosomes were detected in 19 (52.8%) of 36 subjects. Gains of chromosome 3 were more frequent, followed by chromosomes 11, 7, and 18. Thus, the MGUS condition already shows the chromosomal alterations of a plasma cell malignancy [9]. This result indicates that other factors, including alterations of the microenvironment, are likely to be responsible for disease progression [10,11,12]. The processes implicated in the multistep pathogenesis of monoclonal gammopathies are probably extremely composite and involve viruses; adhesion molecules; cytokines; oncogenes including Rb, p53, *ras*, and *bcl*-2; and possibly several, though yet unclear, elements that operate in diverse stages of B-cell maturation. For instance, a transformation cause could be an augmented production of B-cell growth factors.

The B-lymphocyte stimulator (BLyS), which is a tumor necrosis factor (TNF) family member essential for the preservation of normal B-cell expansion, stimulated the survival of malignant B cells. BLyS and its receptor were largely expressed on peripheral and bone marrow B cells in MM subjects compared to those with MGUS and healthy subjects. Additionally, serum BLyS concentrations of MM subjects were higher than those of MGUS subjects and controls [13].

McNee et al. reported a process by which the augmented expression of peptidyl arginine deiminase 2 (PADI2) by bone marrow mesenchymal stem cells (BMMSCs) in subjects with MGUS and MM is clearly modified to a malignant plasma cell phenotype. They recognized PADI2 as one of the most greatly upregulated transcripts in BMMSCs from both MM and MGUS subjects, and that PADI2 provokes the upregulation of interleukin-6 expression. Moreover, they reported a new mechanism by which BMMSC alteration in subjects with MM and MGUS increases pro-malignancy signaling by the citrullination of histone H3R26 [14].

It has also been speculated that the acquisition of CD56 (NCAM) expression in MM is a malignancy-related phenomenon. CD56, a cell adhesion molecule, is highly expressed on MM plasma cells, but is not reported on normal plasma cells [15]. High CD56 expression was described in almost all patients with untreated MM, but in none of the MGUS patients [16,17,18].

### 1.3. MGUS Progression and Immunosurveillance

Escape from immunosurveillance through immunoediting is a hallmark of cancer, and the institution of antitumor immunity necessitates the collaboration of different cell types, including, among others, T cells [19]. Despite the B-cell nature of the condition, significant data demonstrate the crucial contribution of T-cell regulatory activities in the production of the activated B-cell clone in monoclonal gammopathies. The B-cell clonal progenitor that provokes MGUS and myeloma plasma cells displays an Ig class switch and a somatic hypermutation of immunoglobulin heavy-chain-variable (Vh) genes, which are events controlled by T cells [20]. Moreover, several lines of data indicate that the B cell clonal progenitor in MGUS continues to hypermutate Vh region genes, leading to oligoclonal expansion. MGUS and MM subjects also reveal augmented frequencies of T cells specific for the idiotype (Id) of the monoclonal paraprotein that is found at high levels in the sera of these subjects [21,22]. This statement proposes the possibility of an idiotype-specific feedback activation of B cells in a long-lasting stimulatory loop. However, in this regard, it must be recognized that conflicting results are present in the literature, especially for patients with multiple myeloma. In fact, the presence of idiotype-specific Th1 cells and cytotoxic T cells (CTLs) has been interpreted as a defense mechanism which may limit MM development. Previous studies have shown that Id-specific CTLs are able to lyse myeloma cells [23]. Indeed, attempts to vaccinate patients with their own idiotypes have been pursued in clinical trials [24,25].

Several studies have also confirmed the hypothesis that cytokines and lymphocyte activity may predispose to cancer. In a previous study, we focused on the action of cytokines in MM [26]. In this review, we focus our attention on the equilibrium between the diverse lymphocyte subsets and the cytokine system by assessing the action of cytokines and their impact on MGUS. Therefore, the aim of our research was to summarize what is known about this topic to date, providing an overview of the articles examining the role of the entire system in the onset and progression of MGUS.

### 1.4. Immune Paresis and MGUS

Identified risk factors for disease evolution, from MGUS to MM, comprise the amount and type of M-protein, and the ratio of free light immunoglobulin chains. However, several researchers have demonstrated that the suppression of polyclonal immunoglobulin (PIg) production is a relevant progression factor. In a recent paper, the authors compared PIg concentrations with uninvolved heavy/light chain pair (HLC) concentrations to evaluate the degree of immune paresis depending on the MGUS risk category (0–3). They analyzed 159 MGUS subjects who were divided into four risk groups (0—low, 1—low-intermediate, 2—high-intermediate, and 3—high risk of transformation). The study data demonstrated that the degree of MGUS augmentation increases the risk of identified immune paresis through a decrease in PIg levels [27].

Other studies have demonstrated that the evolution to symptomatic MM from MGUS is connected to immune paresis. In fact, the reduction of any IgM isotypes in subjects with IgG or IgA gammopathy, or any IgA isotypes in patients with IgG or IgM gammopathy, was associated with a more rapid time to progression to MM [28].

However, immune paresis seems to depend on several factors. For instance, immune paresis and MGUS are disassociated in advanced age. It was demonstrated that immune paresis peaks with advancing age, but decreases among very old subjects [29].

In any case, the relationships between alterations of the concentrations of cytokines detected in patients with MGUS, the condition of immune paresis, and the onset of infections, are much more complex than they may seem. Immune paresis induced by the dysregulation of cytokines is able to induce infections [30], but the infections could have a major effect on cellular and cytokine status. Then, are the changes reported in cytokine amount secondary to the defects in immune surveillance, to the proliferation of the clonal plasma cells, or to the infections? Ascertaining the connection between the grade of immunosuppression and the degree of MGUS risk would surely influence our comprehension of the malignant evolution of MGUS.

### 1.5. Cellular Subsets

**Figure 1:**
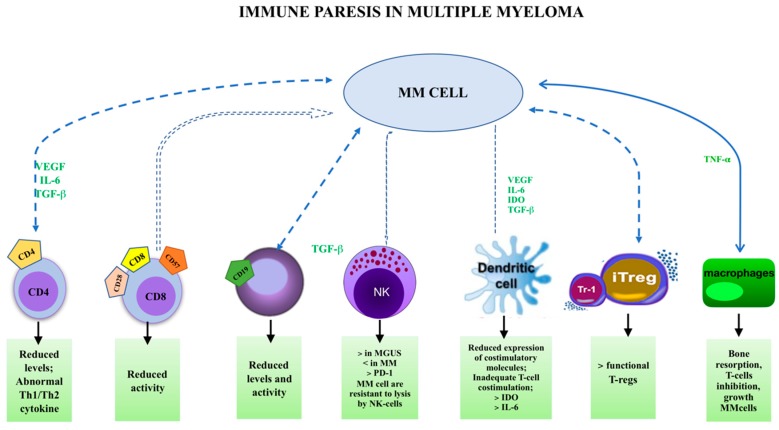
Immune cells involved in multiple myeloma (MM) pathogenesis. The more the arrows are thicker, the most the pathways seems being involved.

A greater part of the studies concerning the bone marrow (BM) microenvironment in MGUS and MM subjects have concentrated their attention on the evaluation of stromal cell interactions, which deliver growth and survival signals to plasma cells [31,32]. Conversely, little consideration has been dedicated to the specific action of peripheral lymphocytes and lymphocytes infiltrating the BM microenvironment [33,34,35] (Figure 1).

Previous reports have demonstrated a reduced number of CD4^+^ T cells in the peripheral blood (PB) of MM subjects, which has been connected to a reduced CD4^+^/CD8^+^ T-cell ratio [36]. Moreover, oligoclonal, chronic growth of circulating T cells, which may involve up to 25% of all T cells, has been reported in the greater part of MM and MGUS subjects [37,38,39]. In some reports, the increase was limited to CD8^+^ T cells exhibiting an activated cytotoxic phenotype (CD8^+^CD57^+^CD28^−^perforin^+^) whereas, in other studies, they were also associated with the proliferation of CD4^+^ T cells that exhibited an effector phenotype (CD28^−^CD4^+^). The specific T-cell increases in subjects with MM have been connected to a favorable prognosis. It is possible that such augmented T-cell populations could be an effort by the immune system to regulate plasma cell (PC) overgrowth [40,41,42].

Perez-Andres et al. demonstrated an augmented rate of BM infiltration by T cells in all subjects with MGUS, MM, and plasma cell leukemia. On the contrary, there was a reduction of CD4^+^CD8^−^ and CD4^−^CD8^−^ T lymphocytes. Similarly, CD4^+^CD28^−^ and CD8^+^CD28^−^ cytotoxic/effector T-cell subsets were also reduced, with an increased expression of TCR-V*b* in both CD4^+^ and CD8^+^ BM T cells in most of the patient groups [43].

In MGUS subjects, specific T-cell populations seem to be augmented. The CD30^+^ T-cell subset and concentrations of CD30 expression are increased in activated lymphocytes from healthy subjects over 60 years of age and in MGUS patients, when compared to younger controls (<60 years). Peripheral blood lymphocytes (PBLs) from MGUS subjects and age-matched healthy controls revealed similar concentrations of IL-6 when stimulated with anti-CD3 plus IL-2, and co-stimulation with a soluble form of the CD30 ligand (sCD30L/CD8a) increased anti-CD3-inducible IL-6 production equally in both groups. Nevertheless, MGUS PBLs also delivered IL-6 when stimulated with sCD30L/CD8a alone. This ability was associated with the presence of CD30^+^ T cells in the peripheral blood of MGUS subjects. Moreover, a greater number of MGUS T cells present the CD30 antigen after activation by incubation with idiotype-expressing autologous serum with respect to those triggered by anti-CD3 plus IL-2. These data indicate that numerical modifications in CD30^+^ T cells are typical of MGUS and aging, and that these cells may influence the chronic stimulation of B cells [44].

Beyond the variable rates of cells present in different situations, diverse activity of the different cell subsets could also be relevant for the progression of MGUS into MM.

The central question is why CD8^+^ T cells fail to regulate the clonal proliferation of transformed plasma cells in MM [45,46,47]. An answer to this problem could be the functional characteristics of CD8^+^ T cells from MGUS and MM subjects, featuring contradictory data. Some studies reported that MM CD8^+^ T cells require protracted in vitro stimulation to produce an effector action, whereas MGUS CD8^+^ T cells show relevant ex vivo activity for autologous plasma cells and MM-associated antigens [48]; these results suggest that MM CD8^+^ T cells are functionally compromised [49]. Conversely, other research has reported that MM CD8^+^ T cells had adequate reactivity against a human leukocyte antigen (HLA)–A2-restricted tumor-associated antigen peptide [50]. An alternative reason why MM CD8^+^ T cells fail to stop tumor progression from MGUS to MM could be that neoplastic plasma cells are altered in the normal presentation of tumor antigens essential for T-cell identification.

These remarks have renewed interest in the immunosurveillance processes of MM growth [51].

Racanelli et al. conjectured that the transformation of MGUS into MM is due to modified plasma cells evading detection by T cells because of altered antigen processing and presenting machinery (APM) [48]. In fact, immunofluorescence and flow cytometry demonstrated significantly diverse patterns of APM component expression in plasma cells from controls, MGUS, and MM patients. A real-time polymerase chain reaction (RT-PCR) demonstrated that APM changes occurred at the transcriptional level. Cytotoxicity assays revealed that in comparison with MM CD8^+^ T cells, MGUS CD8^+^ T cells caused lysis of a greater number of autologous transformed plasma cells. MGUS transformation directly correlated with calreticulin, tapasin, and calnexin expression levels, and indirectly correlated with LMP2 and LMP10 expression levels; MM status did not correlate with APM levels. APM modifications may allow transformed plasma cells to escape immunosurveillance in the MGUS–MM transformation [52]. It was also demonstrated that the antitumor CD8 T-cell action in the BM of MM subjects was less effective than that of MGUS patients [33,34].

However, several reports have attempted to verify if specific subpopulations capable of generating diverse cytokines could distinguish subjects with MGUS from those with MM.

According to the cytokines produced, CD4^+^ T cells can be classified into numerous subsets, including T helper 1 (Th1), Th2, Th17, and CD4^+^CD25^+^ T regulatory (T_reg_) cells.

Th1 cells deliver interferon gamma (IFN-γ) and increase the cell-mediated immune response, while Th2 cells produce IL-4 and reduce the Th1 cell-mediated response. Th1/Th2 polarization is determined by numerous genetic and environmental elements and, particularly, by the local levels of cytokines, such as IL-4 and IL-12, that cause the differentiation of naive T lymphocytes to the Th2 and Th1 phenotype [53]. An association between the type 1 immune response and anticancer activity has been proposed [54,55,56].

The presence of T-cell subsets was analyzed in MGUS subjects and in MM patients with clinical stage I or stage II/III disease. A total of 8 of 9 MGUS subjects, 7 of 12 MM stage I patients, and 3 of 9 patients with MM stage II/III had T cells producing IFN-γ and/or IL-2 (T helper (Th1) type-1 cells), while cells producing both Th1 and Th2 or Th0 types of cytokines were more common in subjects with MM, especially in those with MM stage II/III. The number of Th1-type cells was significantly greater in MGUS subjects compared to those of MM stage II/III [22].

Th17 cells produce IL-17A, IL-6, and TNF-α that are involved in increasing the inflammatory response. T_reg_ cells reduce effector T-cell growth by generating TGF-β and IL-10, which exert immunomodulatory activities. An imbalance between T_reg_ and Th17 cells has a central role in inflammatory diseases.

Recently, Th17 cells have been implicated in the onset of MM and its complications. The CD4^+^ Th1 and CD4^+^ Th17 subsets in patients with MM were significantly higher than those in controls, as were the concentrations of T-bet and RORgamma mRNA [57].

T_reg_ cells have a crucial action in the protection of self-tolerance and the response against tumor cells. An altered T_reg_ action in MM patients could, on the other hand, contribute to MM-related immune dysfunction [58].

The activity of T_reg_ cells in MM patients has been investigated in numerous studies. However, several in vitro or in vivo results remain unclear. For instance, one study evaluated the number of T_reg_ cells in the PB of control subjects versus MGUS and MM patients, and revealed a relevant reduction in the number of T_reg_ cells. Moreover, these cells were incapable of reducing the growth of T lymphocytes. However, a different study assessed the function and number of T_reg_ cells in the PB and BM of controls and MM patients, and did not demonstrate an alteration in the rate of T_reg_ cells [59,60,61].

Numerous processes could counteract dysregulated T_reg_ activity. Indoleamine 2,3-dioxygenase 1 (IDO1) is a tryptophan-catabolizing enzyme. IDO1 oxidizes tryptophan into *N*-formylkynurenine, which is quickly changed to kynurenine (KYN) by the activity of KYN formamidase [62]. KYN reduces effector T cells and increases regulatory T_reg_ differentiation. Bonanno et al. examined IDO1 expression in subjects with symptomatic MM and in subjects with MGUS or SMM. KYN was augmented in 75% of subjects with symptomatic MM and correlated with the increase of CD4^+^CD25^+^FoxP3^+^ T_reg_ cells and the reduction of NY-ESO-1-specific CD8^+^ T cells. Both the T_reg_ increase and reduction of Th1 differentiation were reverted by d,l-1-methyl-tryptophan, an inhibitor of IDO. These results suggest that IDO1 expression may participate in immune suppression in MM patients [63].

Finally, Vγ9Vδ2 T cells are non-conventional T cells halfway between adaptive and innate immunity with the capability to react to tumor cells, comprising malignant MM cells. These cells are capable of recognizing and eliminating MM cells in vitro, but they are blocked in the MM microenvironment by immune suppressive circuits. In fact, bone marrow MM Vγ9Vδ2 T cells are positive for PD-1 and do not respond to phosphoantigen stimulation; remarkably, the PD-1 blockade formed by a single drug is inadequate to completely retrieve their antitumor action in vitro, signifying that other factors are implicated in the anergy of Vγ9Vδ2 T cells. These cells may also play a relevant role in the control of MGUS [64].

An increase in CD4^+^CD25^high^ T_reg_ cells within the blood and the neoplastic microenvironment is a characteristic of tumors. Moreover, increases in T_reg_ cells have been linked to prognosis, tumor stage, and survival. An increased pool of CD4^+^CD25^high^ T_reg_ cells in MGUS subjects and MM patients has also been described. In healthy subjects, low-level expression of CD127 on T cells, in addition to the expression of FOXP3, has been associated with T_reg_ cell increase [65]. Beyer et al. demonstrated that the increased FOXP3^+^ T-cell populations in subjects with MGUS and MM are exclusively CD127^low^ T_reg_ cells, and that they are intensely suppressive. A significant percentage of CD127^low^FOXP3^+^ T_reg_ cells presented only low concentrations of CD25, indicating that the previously described increase of CD25^+^ T_reg_ cells was an underestimate of the actual increase [66]. Since MGUS subjects already have augmented frequencies of T_reg_ cells, it is very probable that the increase of T_reg_ cells is an early process in the onset of MM. Increased levels of T_reg_ cells might be linked to the transformation from the premalignant condition, that is still under control of the immune system, to the proliferation of malignant plasma cells.

In conclusion, the specific effector activities of tumor-infiltrating T cells drive the disease towards regression or progression, according to the diverse T-cell subsets [67,68]. Actually, Th1 cells exhibit antitumor activity, while Th2 and T_reg_ cells are essentially pro-tumor cells [69,70]. What is more questionable is the action of Th17 cells that is determined by the cancer model and tumor microenvironment (Figure 1).

### 1.6. MGUS and Bone Marrow Cell Populations

In order to provide a more comprehensive scenario, we report the changes seen in other cell populations in patients with MGUS and MM.

Key cellular elements of both the normal and transformed bone marrow niche are BMMSCs. There is considerable evidence to suggest that BMMSCs from MGUS and MM patients are phenotypically diverse compared to those derived from control subjects [71,72,73,74,75,76]. Moreover, the BM microenvironment is altered by the malignant plasma cells. An augmented number of macrophages/monocytes has been demonstrated in the BM of MM subjects compared to healthy controls. Tumor-associated macrophages (TAMs) are generally divided into two diverse subtypes: M1 TAMs (tumoricidal) and M2 TAMs (immunosuppressive). M2 TAMs are involved in the generation of a medullary environment with immunosuppressive activity by angiogenesis through vascular endothelial growth factor (VEGF) generation. These M2 TAMs have been demonstrated to augment in MM bone marrow and this increase is linked to poorer survival [77,78].

Plasmacytoid dendritic cells are augmented in the BM of MM subjects and promote Th22 cell differentiation. Di Lullo et al. reported that the number of IL-22^+^IL-17^−^IL-13^+^ T cells is considerably augmented in peripheral blood and BM of stage III and relapsed/refractory MM subjects with respect to control subjects and subjects with asymptomatic or stage I/II disease. Th22 cells cloned from the BM of MM subjects were CCR6^+^CXCR4^+^CCR4^+^CCR10^−^ and delivered IL-22 and IL-13, but not IL-17. A portion of MM cell lines and primary tumors aberrantly exhibited IL-22RA1 and IL-22-induced STAT-3 phosphorylation, cell proliferation, and resistance to drug-induced cell death in MM cells. IL-13 treatment of normal BM mesenchymal stromal cells caused adhesion molecule upregulation, STAT-6 phosphorylation, and augmented IL-6 delivery, and drastically promoted MM cell proliferation compared with untreated BM mesenchymal stromal cells [67].

### 1.7. Inflammation, Cytokines, and MGUS

That an imbalance of T-lymphocyte subsets may play a relevant role in MM [79,80] and an imbalanced pattern of cytokine production by circulating PB T cells was demonstrated in both MGUS and MM subjects [81].

Several chemokines and cytokines have been implicated in the crosstalk between the bone marrow stroma and plasma cells. Interleukin (IL) production by the cells of the BM microenvironment has been demonstrated to play a role in the malignant plasma cell phenotype; firstly, by augmenting the resistance to cell death stimuli, and secondly, by downregulating differentiation markers [82,83,84,85,86].

Chronic cancer-associated inflammation is demonstrated in hematological malignancies, especially in MM, and a diverse cellular and humoral pattern associated with this phenomenon could have an essential role in the onset and progression of monoclonal gammopathies.

In fact, it is known that cytokines are involved in both inflammatory and anti-inflammatory mechanisms, and are part of a system that comprises genes and polymorphisms. Several of these elements that are modified in the serum, or in the BM, of MM patients have proinflammatory actions, such as IL-1, IL-6, IL-12, IL-15, IL-16, IL-17, IL-18, IL-22, IL-23, TNF-α, and IFN-γ, while others have anti-inflammatory actions, such as IL-1Rα, IL-4, IL-10, IL-11, TGF-β1, heat shock proteins (HSPs), and lipoxin A4.

MM is classically exemplified by an altered cytokine system with augmented concentrations of inflammatory cytokines.

In MGUS and MM, chronic inflammation is able to modify the configuration and the function of the monoclonal (mc) Ig generated by the clonal plasma cells by the sialylation of Ig fragment crystallizable (Fc) region. Bosseboeuf et al. studied the sialylation of purified mc and polyclonal (pc) IgGs from MGUS and MM subjects, in comparison to pc IgGs from healthy controls [87]. The inflammatory condition of subjects was evaluated via the dosage of 40 inflammation-linked cytokines. While pc IgGs from MGUS and MM subjects demonstrated heterogeneity in sialylation, mc IgGs from both MGUS and MMM subjects revealed very low sialylation. Furthermore, mc IgGs from MM subjects were less sialylated than mc IgGs from MGUS subjects. Concerning inflammation, 14 cytokines were equally increased in MGUS and MM compared to healthy subjects. MM diverged from MGUS by greater concentrations of IL-11, RANTES, hepatocyte growth factor (HGF), and stroma cell-derived factor 1 alpha (SDF-1-α). MGUS and MM subjects exhibiting hyposialylated pc IgGs had considerably greater concentrations of IL-6, IL-17, IL-33, TGF-β1, HGF, and tumor necrosis factor-α compared to subjects with hypersialylated pc IgGs.

Moreover, MM endothelial cells (ECs) express more HGF and activated cMET versus MGUS ECs and control ECs. The HGF/cMET pathway controls numerous functions of EC in MM subjects, comprising motility, adhesion and, ultimately, the angiogenetic process as a whole. Suppression of this pathway can modify these actions when the inhibition occurs simultaneously with the administration of drugs such as bortezomib or lenalidomide, both in vitro and in vivo. It therefore appears evident that an autocrine HGF/cMET loop supports MM angiogenesis and that this could be a novel target to control angiogenesis in MM patients [88].

Analogously, SDF-1 (CXCL12), a chemokine that controls several systems associated with MM malignant evolution, acts through the specialized receptor CXCR4, which is present on the membrane of MM cells. SDF-1-α regulates the homing of plasma cells in MM patients [89].

In a previous manuscript, we analyzed the inflammatory and anti-inflammatory systems by evaluating the actions of cytokines and their impact on MM [26]. In this section of our review, we have summarized the evidence available regarding this equilibrium investigation of the action of inflammatory and anti-inflammatory cytokines in the onset and progression of MGUS.

### 1.8. Inflammatory Cytokines and MGUS

**Figure 2:**
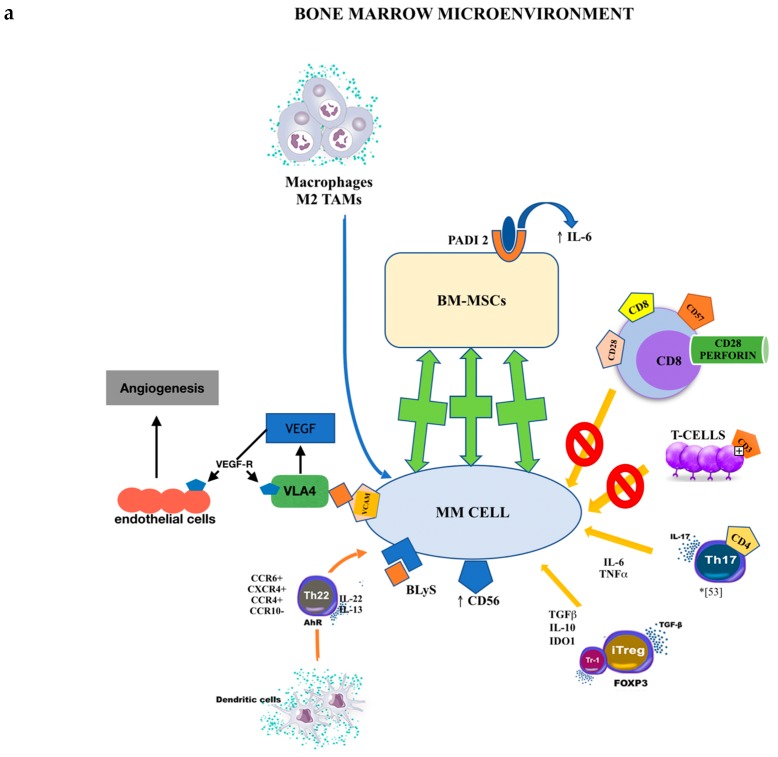

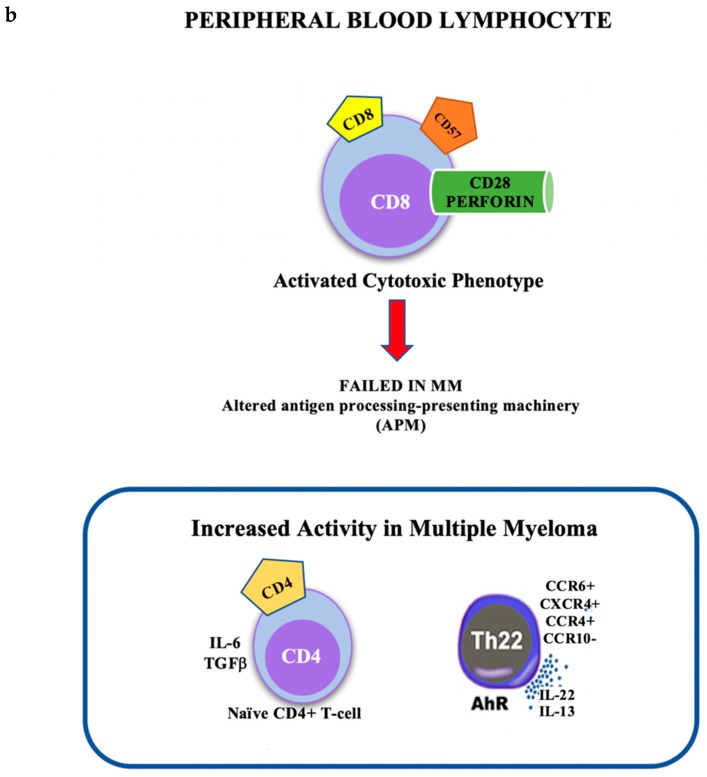
Cytokines and cell subsets involved in the multiple myeloma (MM) peripheral microenvironment. (**a**). Cytokines and cell subsets involved in MM bone marrow microenvironment. (**b**). Cytokines and cell subsets involved in MM peripheral microenvironment.

IL-1 promotes the growth of myeloma cell lines and indirectly operates on myeloma plasma cells through IL-6 generation via a prostaglandin E_2_ (PGE_2_) loop [90]. Normal plasma cells do not deliver IL-1b; nevertheless, abnormal IL-1b generation by myeloma cells has been demonstrated at both the mRNA and protein levels in numerous reports. Employing myeloma cells, Lichtenstein and colleagues [91] identified IL-1b at the protein level, and Klein et al. [92] highlighted strong IL-1b gene expression by in situ hybridization. Cozzolino et al. demonstrated that culture supernatants of plasma cells from all 12 subjects with MM contained high concentrations of IL-1b [93]. On the contrary, plasma cells from MGUS subjects had undetectable concentrations of IL-1b. By employing flow cytometric sorting to enrich plasma cells and RT-PCR for cytokine expression, Donovan et al. confirmed that IL-1b mRNA is produced by plasma cells from all MM subjects but is not measurable in the plasma cells of most MGUS subjects [94] (Figure 2a,b).

Under such analysis, a question arises: Could anomalous IL-1b production be participating in the transformation from MGUS to myeloma?

Hawley et al. employed an animal model of myeloma that demonstrates the relevance of IL-1 expression in provoking a disease that mimics human MM [95,96]. Employing a method that uses retroviral-mediated gene transfer, the authors inserted IL-1 cDNA into an IL-6-dependent B-cell system. After the administration of these IL-1-producing B cells into syngeneic mice, these cells were shown to “home” to the BM and cause lytic bone lesions [96]. Another report has demonstrated that the anomalous expression of IL-1 can modify adhesion molecules, such as ICAM and CD44, on the surface of mouse plasmacytoma cells [97]. An analogous process may happen in human myeloma, in which anomalous expression of IL-1b provokes augmented expression of adhesion molecules, such as CD54, CD56, CD44, and VLA-4, on monoclonal plasma cells [98,99,100,101].

The etiology of acquired IL-1b expression in MM is unclear. However, the IL-1b gene is extremely inducible, and its expression can be modified by several cellular and microbial elements [102]. The action of Kaposi’s sarcoma-associated herpesvirus (KSHV) in the onset of MM has been described [103]. Moreover, Epstein–Barr virus, human immunodeficiency virus-1, and respiratory syncytial virus have been demonstrated to upregulate IL-1 expression either directly by acting with genomic sequences or indirectly by modifying concentrations of transcription factors implicated in IL-1b expression [104,105,106].

Serum and urinary concentrations of the soluble IL-2R (sIL-2R) were considerably augmented in MM subjects compared to healthy subjects, and this increase was directly correlated with disease activity. MM subjects with active disease had considerably greater concentrations than those with stable disease. Compared to healthy subjects, enriched B-cell (but not T-cell) cultures from peripheral blood mononuclear cells (PBMCs) demonstrated significantly augmented amounts of IL-2R+ cells in MM and MGUS. However, the highest amounts were described in active MM compared with stable MM and MGUS. Moreover, 16% of all MM subjects, as opposed to 9% of MGUS subjects, had well-defined BM IL-2R+ plasma cell populations. The results suggest that an alteration of the IL-2/IL-2R system is more evident in active MM than in MGUS [107].

Concerning IL-3 activity in MM subjects, this cytokine promotes the growth of myeloma plasma cells in vitro, but it is essentially implicated in the differentiation of peripheral blood nonadherent mononuclear cells into mature plasma cells [108].

MM subjects have greater serum IL-3 concentrations than those with MGUS. Moreover, IL-3 differentiates PBMCs from MM, but not MGUS, subjects into mature plasma cells [108].

In monoclonal gammopathies, IL-6 has been recognized as a crucial growth factor for human myeloma cells [37]. IL-6 serum concentrations have been correlated with tumor mass and prognosis in MM patients.

A greater proportion of myeloma cells do not generate IL-6 in vivo, but a minority that do produce autocrine IL-6 have been reported [109]. The principal source of IL-6 is paracrine production by BM stromal cells [110,111,112]. An augmented proportion of T cells producing IL-6 were described in MM subjects with active disease (at diagnosis and relapsing) with respect to subjects in remission or with MGUS. Evaluation of serum IL-6 concentrations revealed that the altered IL-6 production by T cells and defective antitumor Th1 cell action were correlated with increased concentrations of IL-6. In vitro experiments of PHA- and anti-CD3/anti-CD28 monoclonal antibodies (MoAbs) stimulation of PBMCs demonstrated the capability of lymphocytes from MM subjects to differentiate into the Th1 subset in the presence of rIL-12. On the contrary, adding exogenous rIL-6 altered IFN-γ production by rIL-12-prompted T cells. The inhibition of Th1 polarization of the immune response by IL-6 occurred directly on T cells and was not mediated by dendritic cells. Assessment of the ability of MM-derived dendritic cells (DCs) to promote the cell growth of allogenic T lymphocytes and produce IL-12 in vitro, in fact, indicated that MM-derived DCs were functionally active. Taken together, these results suggest that augmented IL-6 production by peripheral T lymphocytes influences the immune dysfunction described in MM and allows myeloma cells to escape immune surveillance by inhibiting the antitumor Th1 immune response [113].

A correlation between serum concentrations of IL-6 and the different status of monoclonal gammopathies has been reported by several works. Thus, serum concentrations of IL-6 are augmented in subjects with symptomatic MM compared to subjects with asymptomatic disease and MGUS. Moreover, the highest concentrations were demonstrated in subjects with an advanced disease refractory to treatment [114]. The percentage of MGUS subjects with an increased serum IL-6 concentration ranged from 15% to 35% [115].

Bataille et al. stated that only one out of their 22 subjects with MGUS had an increased serum IL-6 concentration, and this was the only subject where evolution into MM occurred after two years of follow-up [116].

Papadaki et al. described augmented serum levels of IL-6 and soluble IL-6 receptor (sIL-6R) in 48% of subjects with MM, while normal sIL-6R levels were described in a greater number of subjects with MGUS [117]. However, Soutar et al. described augmented serum concentrations of IL-6 in subjects presenting with both MGUS and MM [118].

These discrepancies may be due to the diverse techniques that have been employed to assess IL-6 activity. Moreover, IL-6 concentrations exhibit daily diurnal (morning and afternoon) modifications [119]. IL-6 has a short half-life, thus, serum concentrations inadequately correlate with local production and only reproduce the part not bound to cellular receptors. For this reason, a single evaluation of serum IL-6 should be treated with caution.

In an effort to longitudinally study the action of serum IL-6 levels in MGUS subjects and to establish the predictor significance of this cytokine in the transformation from MGUS to MM, Blade et al. compared the IL-6 concentrations of MGUS patients to those of controls. After a median follow-up of seven years from the initial cytokine assessment, nine MGUS subjects had progressed to MM. However, the actuarial probability of evolution in MGUS subjects with augmented IL-6 concentrations was not significantly higher than in those with normal levels [120].

Furthermore, several studies have considered the clinical significance of soluble IL-6 receptor levels (sIL-6R) in subjects with plasma cell dyscrasias, with similar results. The sIL-6R concentrations in malignant gammopathies are higher than in MGUS [117,121].

However, in another report, among healthy subjects and MGUS and MM patients, sIL-6R levels were augmented analogously in MGUS and MM [122].

Finally, IL-6 activity in normal plasma cells (nPCs) and abnormal plasma cells (aPCs) is CD126- (subunit of IL-6 receptor) dependent. Salem et al. measured CD126 expression on nPCs and aPCs in MGUS, SMM, and MM. CD126 was identified on all nPCs and aPCs. However, CD126 expression was higher in aPCs than in nPCs in 85% of SMM, but only 41% of MGUS. There was also a greater correlation between nPC and aPC CD126 expression in low-risk MGUS than described in high-risk MGUS and SMM, indicating a reduction in normal regulation of CD126 with disease transformation [123].

IL-12 concentrations seem to be altered in MM subjects compared to MGUS subjects.

A group of PB myeloid cells expressing the MDC8 antigen having a 6-sulfo LacNAc structure and named 6-sulfo LacNAc dendritic cells (Slan-DCs), has been reported. Slan-DCs form a tissue and circulating pro-inflammatory myeloid population which has been demonstrated to have a role in different tumor environments. Lamathee et al. investigated Slan-DCs in subjects with monoclonal gammopathies [124]. They executed functional research on the cells of newly diagnosed MM patients, MGUS subjects, and MM patients who responded to therapy. They observed that circulating Slan-DCs were considerably reduced in MM subjects compared to those of healthy controls or MGUS patients. Moreover, after activation by the TLR7/8 ligand R848, IL-12-producing Slan-DCs from the BM or PB from MM patients were reduced compared with healthy controls, and MM cells reduced the generation of IL-12 by Slan-DCs. Moreover, they modified the phenotype of Slan-DCs towards that of a monocyte-like phenotype. Lastly, they reported that Slan-DCs co-cultured with MM cells decreased their ability to cause T-cell growth and Th1 polarization.

IL-17, a disulfide-linked homodimer generated by activated memory T cells, is thought to be a mediator between the hematopoietic and immune systems, and promotes the production of IL-6 and Intercellular Adhesion Molecule (ICAM). The production of IL-17 in the BM of MM patients by T-helper lymphocytes is mediated by dendritic cells. Unlike in MGUS, the BM of MM patients contains a high number of Th-17-1 cells with the co-expression of 1L-17 and IFN-γ, interrelating with apoptotic myeloma elements [125]. However, in spite of these results, the study demonstrated no modifications in the serum concentrations between MGUS and MM.

Inflammation may affect the structure of both pc Ig and mc Ig produced by malignant plasma cells through the sialylation of Ig Fc fragments [87]. MGUS and MM subjects exhibiting hyposialylated pc IgGs had considerably greater concentrations of IL-33 compared to subjects with hypersialylated pc IgGs. In MGUS, as in MM, the hyposialylation of mc IgGs is concomitant with augmented concentrations of cytokines that have a central role in inflammation [87]. However, decreased IL-33 concentrations in MM subjects are associated with a more advanced stage of disease [126].

This result seems to be consistent with the evidence that a decrease of IL-33 might be implicated in T-cell alteration in hematological diseases [127].

Macrophages and monocytes can deliver inflammatory cytokines that increase the proliferation and survival of myeloma cells. Moreover, macrophages produce tumor necrosis factor α (TNF-α), which can indirectly increase tumor proliferation [128,129]. In a study published some years ago, Bladè et al. reported that MGUS subjects with high concentrations of TNF-α had a greater probability of malignant progression than those with low serum concentrations [130].

Finally, although several studies have shown a different concentration of IL-16 in patients with MM, no study has evaluated cytokine concentrations in patients with MGUS [131].

### 1.9. Anti-Inflammatory Cytokines

The existence of a pro-inflammatory or anti-inflammatory action of IL-4 depends on the experimental conditions in which they are verified. The role of IL-4 in MM is also controversial. It can reduce IL-6 production and, consequently, plasma cell growth [132]. On the other hand, it also promotes the differentiation of PBNMCs into mature plasma cells, possibly because PBNMCs are not sensitive to IL-6 action [133,134].

However, the activity of IL-4 on the differentiation of PBMNCs into mature plasma cells is more marked among cells from MM than MGUS patients.

Interleukin-10 is the most powerful B-cell differentiating factor, although it does not appear to operate on myeloma cells [135]. More recently, it has been demonstrated to promote the growth of myeloma cells in IL-6-deprived cultures. Therefore, it can be considered an IL-6-unrelated growth factor for myeloma plasma cells [136].

IL-10 concentration was evaluated in MGUS, in early or advanced stage MM, and in aggressive diseases. The highest number of subjects with measurable IL-10 was reported at early stages, as opposed to those with advanced or aggressive MM. IL-10 was not measurable in MGUS subjects. Thus, IL-10, like IL-6, may have a diagnostic value in differentiating MM from MGUS. However, unlike IL-6, high concentrations of IL-10 are associated with a better prognosis. Regrettably, no clinical studies have confirmed these data. In MM, the cytokine was detected at similar concentrations in all stages of the disease. Furthermore, IL-10 levels did not vary significantly between patients and healthy subjects, nor between MGUS subjects and MM patients [137].

IL-27 activity might differ according to the target cell type. However, IL-27 concentrations seem to be greater in MM subjects with active disease (MMECs) than in MGUS subjects.

Actually, IL-27 has been demonstrated to stimulate PD-L1 expression in several cell types, including CD4^+^ and CD8^+^ T cells, monocytes, DC, and tumor cells [138,139,140] Therefore, IL-27 might cause possible actions regulating the expression in MMECs of HLA-I, which downregulates NK cell activity, and also that of PD-L2, which regulates the level of both CD8^+^ T and NK cell activity. It has been demonstrated that in MM subjects, more than the 50% of PB NK cells express PD-1, the PD-Ls receptor, although at concentrations lower than PD1^+^ in NK cells, as reported in healthy controls or in diverse diseases [141]. The ability of IL-27 to upregulate immune checkpoint ligands on tumor endothelium may significantly differ among patients. Remarkably, throughout the range of antitumor immune responses, IL-27 may operate earlier than IFN-γ, a factor delivered by innate cells such as macrophages and DCs.

Moreover, angiogenesis is characteristic of MM progression. Dondero et al. investigated the action of cytokine-stimulated NK cells against tumor-associated endothelial cells isolated from the BM aspirates of MM subjects with MMECs [142]. They demonstrated that NK cells stimulated with adequate doses of IL-15 destroyed MMECs via the activity of multiple activating receptors. In particular, according to the increased expression of Poliovirus receptor (PVR) and Nectin-2 on MMECs, DNAM-1 actively contributed to target detection. Remarkably, in MMECs, the density of PVR was considerably higher than that reported from MM subjects with MM in complete remission or with MGUS. Notably, IL-27, which unlike IL-15 does not show pro-angiogenic activities, augmented the NK cell functions provoked by suboptimal levels of IL-15.

A preliminary study reported substantially lower IGF-1 concentrations in MGUS and MM subjects compared with healthy controls, with the concentrations being significantly lower in MM than in MGUS [143]. However, in another study, IGF-1 concentrations were only occasionally higher in MGUS than in MM, with considerably higher concentrations in substage B.

Moreover, subjects with MM had drastically lower levels of serum transforming growth factor (TGF)-β than subjects with MGUS [144].

Finally, the molecular chaperone heat shock protein interacts with several client proteins, either intracellular or cell-surface localized, which are functionally implicated in several essential regulatory pathways, such as cell cycle control and protection from apoptosis. Furthermore, its action appears to be indispensable for cancer cells in supporting abnormal homeostasis [145].

Small ubiquitin-like modifier (SUMO) belongs to a group of small proteins that modify the functions of target protein binding to amino acid residues [146,147,148]. Sumoylated proteins are implicated in several cellular mechanisms, including DNA damage response, response to stress, protein stability, transcriptional regulation, cell proliferation, and apoptosis [149,150,151].

**Figure 3:**
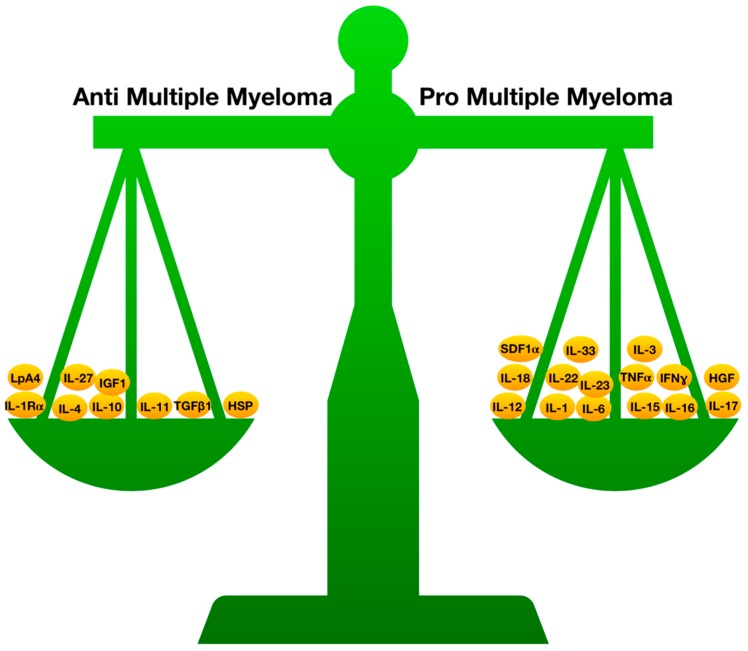
Balance of pro- and anti-inflammatory cytokines in multiple myeloma (MM) progression.

Preuss et al. recognized a post-translationally-modified paraprotein target (paratargs) in MGUS and MM [152]. They reported that paraproteins from some subjects reacted with the sumoylated heat shock protein 90 β isoform-α (HSP90-SUMO1), while no effect was demonstrated in healthy controls. HSP90-SUMO1 was discovered in blood cells from all patients with HSP90-SUMO1-binding paraproteins. They established that the HSP90-SUMO1 carrier state is provoked by the incapacity of SUMO peptidase sentrin/SUMO-specific protease 2 (SENP2) to desumoylate HSP90-SUMO1. Although HSP90-SUMO1 was reported in a small percentage of controls, only MGUS and MM subjects who were HSP90-SUMO1 carriers produced HSP90-SUMO1-specific paraproteins, suggesting that sumoylated HSP90 contributes to the onset of these conditions (Figure 3).

## 2. Conclusions

Almost all MM cases have been demonstrated to be linked to earlier MGUS. Nevertheless, no marker that would consistently foresee the onset of the two diseases with such different prognoses and therapeutic approaches is presently available in clinical practice (Table 1).

Inflammatory cytokines are largely delivered in chronic hematological diseases, but the causes remain indeterminate and are likely to be multiple. Some cytokines may be generated by neoplastic cells as an effect of genetic mutations, but there is solid proof that cytokines are also delivered autonomously from gene mutations, both by clonal and non-clonal cells [154].

Therefore, cytokines can be used as therapeutic targets with several advantages. In fact, cytokines can be reduced, and cytokines are well-studied in animal experimental models employing genetic models such as knockout mice or neutralizing antibodies. On the contrary, emerging therapeutic attempts should not ignore the possible generation of a cytokine storm that might manipulate immune responses against myeloma cells and/or the tumor-associated microenvironment.

However, scientists should learn to recognize which cytokines have to be suppressed and which ones should be strengthened in an attempt to prevent the progression of MGUS into MM.

A model that predicts the negative action of pro-inflammatory proteins and the positive effect of anti-inflammatory proteins appears excessively simplistic and, as previously demonstrated for MM patients, completely inadequate [26].

Indeed, although several inflammatory cytokines appear to be reduced in patients with MGUS compared to those with MM (for example, IL-1, IL-2, IL-3, IL-17, and TNF), others appear to be increased (IL12) or equally expressed (IL-33). Similarly, anti-inflammatory cytokines seem to be both increased (TGF-1) and reduced (IL-4, IL-27, and IGF-1) in MGUS.

Proinflammatory cytokines are supposed to be essential for cancer progression, and anti-inflammatory drugs have been suggested to cure tumors. However, anti-inflammatory treatments may hypothetically decrease protective antitumor immunity. In fact, although inflammation is generally considered to be cancer-supporting, few reports in colorectal, breast, and bladder cancer indicate that tumor infiltration by inflammatory cells may be connected with an improved prognosis [155,156,157].

Pro-inflammatory cytokines can have both pro- and anticancer actions, while cytokines with strong anti-inflammatory action may strongly support the proliferation of cancer cells. To bring together these conflicting results, it is possible to propose that inflammation, when driven by tumor-specific Th1 cells, may stop tumor onset and progression. In a Th1 microenvironment, pro-inflammatory cytokines may participate in cancer eradication by attracting leucocytes from circulation and by augmenting CD4^+^ T-cell action.

In the future, however, other aspects of the action of the immune system on the onset of gammopathies will have to be evaluated. In particular, special attention must be given to the action of alarmins and innate lymphoid cells (ILC).

Alarmins, such as high-mobility group box 1 (HMGB1) and S100 protein (S100), are a responsive set of proteins. They are produced as a result of cell injury, damage, or inflammation. They have several actions as they can trigger innate immunity and stimulate antigen-presenting cells (APCs), activating an adaptive response. However, they could alter homeostasis by causing an excessive pro-inflammatory response [153,158].

The ILC family includes cytotoxic NK cells, ILC1, ILC2, ILC3, and lymphoid tissue inducer (LTi) cells [159,160]. These cells have critical roles in host defense and inflammation, representing the first-line defense against infection. ILCs present great correspondence with several T-cell subsets, suggesting that ILCs are the “innate” form of differentiated CD4^+^ and CD8^+^ T cells [161,162,163,164].

However, in patients with MGUS, interpretation of the role of lymphocyte populations and cytokines appears, in any case, to be more complex and difficult than in MM. In fact, any evaluation of the parameters appears static and inadequate in MGUS. Only longitudinal evaluation of the lymphocyte populations and the cytokine levels, assessed at different times and correlated with the eventual progression of the condition towards MM, will allow for the correct interpretation of data and development of an effective therapeutic approach.

In an age where the therapy of neoplastic monoclonal gammopathies largely relies on drugs capable of acting on the immune system (immunomodulants, immunological checkpoint inhibitors, CAR-T), detailed knowledge of the conditions existing in benign and neoplastic forms of gammopathy is the main foundation for the adequate and optimal use of new drugs. MM is accompanied by cellular immune defects, suggesting that transformation of the disease from a benign, precursor state is connected with an immunosuppressive milieu that promotes immune escape and tumor onset [165]. Nevertheless, the complexity of the BM microenvironment makes it difficult to develop efficacious immune therapies for MM. T cells have been demonstrated to be able to identify tumoral plasma cells (PCs), as demonstrated by the discovery of bone marrow-infiltrating T cells in MGUS capable of producing anti-PC immune responses. Moreover, the presence of immunity against plasma cell antigens is associated with delayed progression to MM. Nevertheless, once MM arises, bone marrow T-cell responses have not been described [166]. The rarity of antigen-specific T cells is one reason that immune checkpoint inhibitors such as anti-PD-1 have inadequate clinical efficacy in MM subjects [167]. Remarkably, lenalidomide seemed to have transitory efficacy after nivolumab during a phase where protracted receptor occupancy of the PD-1 receptor was imagined [168]. In view of the above, it might be interesting to hypothesize a study that attempts to prevent the progression of high-risk MGUS by using checkpoint inhibitors.

In this scenario, biologics represent a fascinating hope for the near future. Anakinra, the recombinant form of the IL-1 receptor antagonist (IL-1Ra), could be used for clinical studies of MGUS patients. IL-1Ra inhibits the binding of IL-1α and IL-1β to IL-1R1. Even though anakinra is currently approved for the therapy of rheumatoid arthritis and cryopyrin-associated periodic syndromes [169], off-label employment of anakinra in several other diseases, and the theoretical possibility of intervening in the progression of MGUS, or even in the therapy of MM with the use of anakinra, certainly appears fascinating. Presently, there are very little data on this topic. However, this molecule has been positively employed for the treatment of complex disorders such as Schnitzler’s syndrome, which is defined by monoclonal gammopathy (IgG or IgM) and a frequent urticaria rash, accompanying clinical symptoms of inflammation and an increased possibility of AA amyloidosis and lymphoproliferative diseases. These subjects are successfully treated with anakinra subcutaneously [170]. However, only the implementation of studies specifically designed to evaluate the effect of the inhibition of IL-1 with anakinra will be able to clarify the real role of cytokines in the onset and progression of MGUS.

To summarize, our review shows that the progression from MGUS to MM is a complex, and not yet completely clarified, process. Literature results have demonstrated that pro-inflammatory cytokines and important anti-inflammatory mediators, such as those represented in Figure 3, are fundamental. Their main function is in the regulation of immune system cells (CD4, CD8, Th17, Treg, Th22, ILC). These cytokines can have both pro- and anticancer actions, while cytokines with a strong anti-inflammatory action may strongly support the proliferation of cancer cells. This fragile balance, if disrupted, could favor MGUS progression to MM. Further studies should consider mediator levels in patients undergoing biologic treatments, in order to better clarify this delicate balance.

## Figures and Tables

**Table 1:** List of the most recent studies (2009–2019) concerning the monoclonal gammopathy of undetermined significance (MGUS) and multiple myeloma (MM) cytokine profiles. Values + = molecules increased. Values − = molecules decreased. BM = bone marrow.

Authors	Year	MGUS	MM	Humans	Animals	In Vivo	In Vitro	Ex Vivo	N. Pz.	Values
Landgren O. et al. [10]	2009	x	x	x		x		x	71	+M protein
Chiecchio L. et al. [11]	2009	x	x	x		x			716	+deletion/monosomy 13 (Δ13)
Garayoa M. et al. [76]	2009		x	x			x		26	distinct genomic profile
Chen H. et al. [77]	2009		x		x	BM	x			+PTN
Dezorella N. et al. [86]	2009		x	x		BM	x	x		−CD38; −CD138
Greco C. et al. [143]	2009	x	x	x		BM	x	x	71 with MGUS; 77 with MM	−IGF-I
Josselin N et al. [153]	2009	x	x	x		BM		x	53 with MGUS; 46 with MM	+dendritic cells; +osteoclasts
Racanelli V. et al. [52]	2010	x	x	x		BM		x	20 with MGUS; 20 with MM	MM → APM components
Bonanno G. et al. [63]	2012	x	x	x		x	x		7 with MGUS; 25 with MM	+IDO activity
Wang P. et al. [13]	2013	x	x	x		BM	x	x	11 for MGUS; 13 for MM	+BAFFR; +TACI; +BCMA in MGUS; −BCMA in MM
Mehtap O. et al. [80]	2014		x	x		BM			44	−IL-21; +IL-6, +IL-1β, +TNF-α
Ferrucci A. et al. [88]	2014	x	x	x		BM	x		24 with MGUS; 32 with MM	+HGF/cMET
Feng P. et al. [60]	2015		x	x		x		x	33	+Th1; +Th17; −T_reg_; +IL-6; +IL17A; +IFN-γ; −Foxp3
Wang M. et al. [61]	2015		x	x		BM		x	55	+Th17; +Th22
Di Lullo G. et al. [67]	2015	x	x	x		BM	x	x	5 with MGUS; 37 with MM	+IL-13; +IL-17; +IL-22
Sponaas A.M. et al. [78]	2015		x	x		BM				+CD14⁺; +CD16⁺
Koerber R.M et al. [89]	2015		x	x			x			−Syk
Nair S. et al. [6]	2016	x	x	x	x	BM		x	20 Gaucher’s disease in humans and 6 GBA 1 mice	+LGL1
Bosseboeuf A. et al. [5]	2017	x	x	x		x		x	101 for MGUS; 135 for MM	+IL-6, + IL-10, +IL-33 in MGUS; −IL-33 in MM
McNee G. et al. [14]	2017	x	x	x		BM	x		30	+IL-6; +CXCL12; +cMET
Bosseboeuf A. et al. [87]	2017	x	x	x				x	68 with MGUS; 74 with MM	+IL-17, +IFN-α2, +IL-33, + IL-13
Dondero A. et al. [142]	2017	x	x	x		BM	x		9	+NK; +IL-27
Salem D. et al. [123]	2018	x	x	x		BM			24 with MGUS; 35 with MM	+CD126
Lamarthée B. et al. [124]	2018	x	x	x		BM	x		21 with MGUS; 24 with MM	−Slan-DC for MM; +Slan-DC for MGUS; -IL-12
Nair S. et al. [7]	2018	x	x	x	x	x		x	cohort 1 (76); cohort 2 (274)	+Ig G; +LPC
